# Investigation of *Mycobacterium paratuberculosis* in Arabian dromedary camels (*Camelus dromedarius*)

**DOI:** 10.14202/vetworld.2019.218-223

**Published:** 2019-02-09

**Authors:** Mohamed A. Salem, Wael M. El-Deeb, Ahmed A. Zaghawa, Fadel M. Housawi, Ahmed M. Alluwaimi

**Affiliations:** 1 Veterinary Teaching Hospital, King Faisal University, Al-Hasa, Kingdom of Saudi Arabia; 2 Department of Medicine and Infectious Diseases, Faculty of Veterinary Medicine, Cairo University, Cairo, Egypt; 3 Department of Clinical Studies, College of Veterinary Medicine, King Faisal University, Al-Hasa, Kingdom of Saudi Arabia; 4 Department of Veterinary Medicine, Infectious Diseases and Fish Diseases, Faculty of Veterinary Medicine, Mansoura University, Mansoura, Egypt; 5 Department of Infectious Diseases, Faculty of Veterinary Medicine, Sadat University, –Sadat, Egypt; 6 Department of Microbiology and Parasitology, College of Veterinary Medicine, King Faisal University, Al-Hasa, Kingdom of Saudi Arabia

**Keywords:** isolation, Johne’s disease, *Mycobacterium avium*, paratuberculosis, polymerase chain reaction

## Abstract

**Aim:**

*Mycobacterium avium* subspecies *paratuberculosis* (MAP) causes Johne’s disease in ruminants. This study aimed to investigate *Mycobacterium paratuberculosis* infection in clinically infected camels on the immunological, conventional bacteriological, and molecular biological basis.

**Materials and Methods:**

A total of 30 Arabian camels (*Camelus dromedarius*) were examined in this study. The camels were suffering from signs ranging from mild to severe infections (that did not respond to antibiotic treatment) to chronic or intermittent diarrhea. Camels were grouped into three groups based on their age, sex, and breed. Detection of anti-MAP antibodies in camels’ serum, Ziehl–Neelsen (ZN) staining technique on rectal scraps, direct recognition of MAP in stool and tissue specimens by IS900 polymerase chain reaction (PCR) assay, and finally isolation and molecular description of MAP from fecal and tissue samples were carried out.

**Results:**

Five MAP isolates were recovered from these investigated camel samples giving an isolation rate of 16.6%, while eight camels were identified by PCR (26.6%). Five camels yielded MAP in their feces by ZN fecal staining (16.6%), whereas ELISA detected anti-MAP antibodies in nine camels only (30%).

**Conclusion:**

From the obtained results, we concluded that the gold standard for the diagnosis of MAP is the culture method despite its limitations. Molecular diagnosis (PCR) could be a useful tool in the identification of truly positive and negative camels; however, great care should be given regarding the primers specificity and sensitivity.

## Introduction

Johne’s disease is one of the most economically important infectious diseases that are caused by infection with *Mycobacterium avium* subspecies *paratuberculosis* (MAP) in ruminants [1]. In the Kingdom of Saudi Arabia, the disease is recognized in ovine, dairy cattle, and dromedary camels [2-5].

The main route of infection in Johne’s disease is through consumption of contaminated food and milk with feces of infected animals. It was previously reported that, during the subclinical infection of cattle, the animal shed a low number of MAP in their feces. Conversely, a dramatic increase in fecal bacterial shedding is noticed in clinically infected cattle. Johne’s disease is manifested by chronic non-responsive diarrhea, leanness, marked reduction in milk production, and infertility in cattle [6]. The clinical and pathological characteristics of MAP in dromedary camels were comparable to MAP in clinically infected cattle [7-13].

The immune response of infected animals against MAP infection is varying to some extent [14]. Regardless of the many research activities on the MAP pathogenesis, the total component by which MAP bolsters its ingenuity and intercedes the immunosuppressive status of the host is as yet restricted.

Previous research efforts showed the existence and a wide spread of MAP infection in the dromedary camel (*Camelus dromedarius*).

Literature available on MAP infection in camels includes a vast hole of data regarding the illness pathogenesis, camel impenetrable reactions to the disease, or components that enhance camel’s protection against MAP [15].

MAP with Ziehl–Neelsen (ZN) stain appears as an intracellular slow-growing bacteria, short, red staining rods, settled in clumps [16]. Conferring to the molecular basis and the colony properties on the culture, the MAP strains were categorized into three types: Sheep, cattle, and intermediate types. The molecular evaluation of MAP strains is based on the restriction fragment length polymorphism (RFLP) analysis combined with hybridization to the insertion sequence IS900 (IS900-RFLP). Moreover, the culture features of MAP are mainly depended on the formation of yellow-orange pigment, colonies shape on solid media, the growth rate and by differences in their cell wall antigen lipoarabinomannan [17-19].

The MAP strain concerned with Johne’s disease in dromedary camels was previously isolated and fully sequenced [20]

This study aimed to investigate *Mycobacterium paratuberculosis* infection in clinically infected camels on the immunological, conventional bacteriological, and molecular biological basis.

## Materials and Methods

### Ethical approval

This study approved by Institutional Animal Ethics Committee of College of Veterinary Medicine, King Faisal University, Al-Hasa, Kingdom of Saudi Arabia.

### Animals

A total of 30 Arabian dromedary camels (*C. dromedarius*) were scrutinized in this investigation. These camels were admitted sporadically or in small groups to the Veterinary Teaching Hospital, College of Veterinary Medicine, King Faisal University, Saudi Arabia, in the period from March 2014 to December 2015 suffering from signs of chronic or intermittent diarrhea that are non-responsive to antibiotic treatment. Animals under investigation were clinically examined according to standard methods [21]. Animals were grouped into three groups based on their age, sex, and breed (Table-1).

**Table-1:** Clinically positive camel (n=30) demographics and results.

Groups	Age (years)	Number of samples	Sex	Number of positive camels				

				Clinical signs	ELISA	ZN^*1^	IS900 PCR	Culture
Group 1	1-3	7	F^*2^=4	7 (F=4, M=3)	0	0	0	0
			M^*3^=3					
Group 2	4-5	9	F=5	9 (F=5, M=4)	2 (F=2, M=0)	0	0	0
			M=4					
Group 3	6 and above	14	F=9	14 (F=9, M=5)	7 (F=5, M=2)	5 (F=3, M=2)	8 (F=6, M=2)	5 (F=3, M=2)
			M=5					
Total		30	F=18	30 (F=18, M=12	9 (F=7, M=2)	5 (F=3, M=2)	8 (F=6, M=2)	55 (F=3, M=2)
			M=12					

*1 Ziehl–Neelsen stain,

*2 Female,

*3 Male, PCR: Polymerase chain reaction

### Samples

Rectal scrapings mixed with feces were collected from each animal according to a previously described method [22] using a long-handled spoon-shaped curette from the rectal wall. Scrapings were sent to the laboratory in disposable plastic cups where they were processed and examined as soon as possible. Blood was collected in collection tubes without anticoagulant and allowed to separate at 5°C overnight. Serum samples were stored at −20°C until further processing.

### Detection of anti-MAP antibodies with ELISA

The existence of MAP antibodies was detected in camel serum samples using the ID Screen^®^ Paratuberculosis Indirect ELISA kit (ID VET diagnostics, Montpellier, France).

### Microscopic examination

ZN staining technique was performed on rectal scraps according to a previously described method [19].

### Direct recognition of MAP in camel feces and tissue specimens by IS900 polymerase chain reaction (PCR) assay

The DNA of MAP was extracted from stool specimens using DNA extraction stool mini kit, and the procedure was carried out with slight modifications to the direction of the manufacturer (Qiagen). This PCR assay was carried out using the primers and PCR conditions designated previously [23].

The primer set used in this PCR was the primer pair TJ1 (5` GCT GAT CGC CTT GCT CAT- 3`) and TJ2 (5`CGG GAG TTT GGT AGC CAG TA - 3`) in the first-round PCR and primer pair TJ3 (5`CAG CGG CTG CTT TAT ATT CC- 3`) and TJ4 (5` GGC ACG GCT CTT GTT GTA GT- 3`) in the nested PCR. In the first-round PCR, the primers were used in a final concentration each of 2 μM in a 50 μl PCR reaction master mix which also included 1.25 U Taq polymerase, 1× PCR buffer, 1.5 mM MgCl2, 200 μM dNTP mix, and 5 µl of isolated DNA. The thermal cycler parameters used for amplification included an initial 95°C incubation step for 10 min for Taq polymerase activation, followed by 30 cycles of denaturation at 95°C for 1 min, annealing at 58°C for 1 min, and extension at 72°C for 2 min with a final extension step at 72°C for 10 min. The amplification products (357 bp) were visualized on an ultraviolet transilluminator after electrophoresis at 125 volts for 45 min in 1.5% agarose gels pre-stained with ethidium bromide. For the nested PCR, 5 µl of the first-round reaction mixture was transferred into a 45-µl reaction mixture that had the same ingredients as the primary reaction mixture but with 2 µM (each) primers from the primer pair TJ3 and TJ4. Cycling conditions were 1 cycle of 95°C for 5 min and then 40 cycles of 95°C for 1 min, 58°C for 1 min, 72°C for 2 min followed by 1 cycle of 72°C for 10 min. Amplicons of the expected size (294 bp) were visualized with ethidium bromide on 1.5% agarose gels.

The entire 417 bp open reading frame of the sequence 251 was amplified using the forward primer 251 F (5- CAC GTG CTG TCC CCA TCG GC -3) and the reverse primer 251 R (5- CTA CGT CTT CGT GAC CAA AG-3). PCR recipes included primers with a final concentration of 0.5 μM (each) in a 20 μl PCR reaction master mix, approximately 50 ng of template genomic DNA (obtained by direct bacterial boiling at 100°C for 10 min) as well as 0.5 units Taq polymerase, 1× PCR buffer, 1.5 mM MgCl2, 200 μM dNTP mix, and distilled water. A positive control (DNA from the 19698 strain) and negative control (sterile bi-distilled water) were included in each PCR run. Amplification parameters for the sequence 251 included an initial 95°C incubation step for 15 min, followed by 35 cycles of denaturation at 94°C for 20 s, annealing at 53°C for 30 s, and extension at 72°C for 30 s with a final extension step at 72°C for 7 min [24].

### Isolation of MAP from camel feces

Samples were decontaminated, and cultured on 3 HEYM slants supplied with mycobactin J and 1 HEYM slant without mycobactin J (Becton Dickinson). After incubation at 37°C for 40 weeks, cultures with visible growth were further processed to identify MAP primarily on the basis of colonial characters, acid fastness, and mycobactin J growth dependence [19,25].

### Molecular identification of isolates

DNA was obtained from visible colonies by direct bacterial boiling at 100°C for 10 min and was used for two specific PCR assays, an IS900 PCR and amplification of the locus 251. The procedure of IS900 PCR was the same as mentioned above, while sequence 251 was amplified using the PCR conditions [19, 24].

## Results

**Figure-1:**
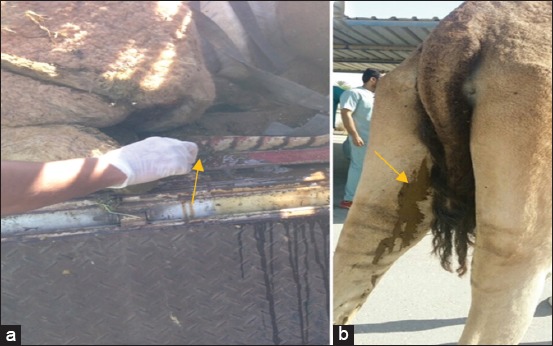
Clinical picture of Johne’s disease. (a) Rectal scrapings in a camel suffered from chronic diarrhea. (b) Diarrhea on a camel hind quarter.

Of 30 camels that suffered from different signs of diarrhea (Figure-1), from mild to severe, non-responsive to antibiotic treatment, to chronic or intermittent, five MAP isolates were recovered from these investigated camel samples giving an isolation rate of 16.6%, while eight camels were identified by PCR (26.6%). Five camels yielded MAP in their feces by ZN fecal staining (16.6%), whereas ELISA detected anti-MAP antibodies in nine camels only (30%).

**Figure-2:**
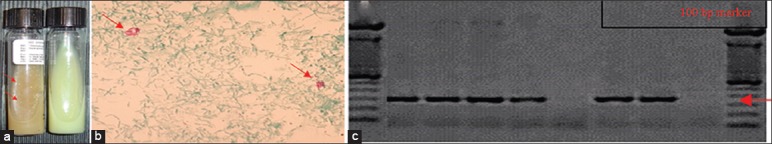
Diagnostic test results. (a) Culture: Small white to yellow half ball shape or pinhead *Mycobacterium avium* subspecies *paratuberculosis* colonies (left side tube), while no colonies were observed on the right side tube. (b) ZN stain: Detection of acid-fast bacilli after microscopic examination. (c) Polymerase chain reaction: Amplification products (357 bp) were visualized on an ultraviolet transilluminator after electrophoresis at 125 volts for 45 min in 1.5% agarose gels pre-stained with ethidium bromide.

After 4-20 weeks of incubation, colony identity was confirmed by colony morphology (the detection of small white to yellow half ball shape or pinhead Map colonies), ZN acid-fast staining, and application of IS900 PCR and amplification of the locus 251 (Figure-2). Two phenotypes were detected, one was described by visible colonies after 16-20 weeks (slow growers), and the other phenotype was characterized by visible colonies after 6-12 weeks (fast growers). This study declared that the incidence of MAP was higher in females than males by all tests. Results are shown in Tables-1 and 2.

**Table-2:** Clinical disease outcomes (ranged from acute to chronic disease) of the camel cases in correlation to the isolation rate by different methods.

Demographics	Number of samples	Number of positives				

		Clinical signs	ELISA	ZN^*1^	IS900	Culture
Age						
Group 1	7	7	0	0	0	0
Group 2	9	9	2	0	0	0
Group 3	14	14	7	5	8	5
Sex						
Male	12	12	2	2	2	2
Female	18	18	7	3	6	3
Clinical outcome						
Subclinical	-	-	-	-	-	-
Mild	7	7	0	0	0	0
Moderate–severe	17	17	4	2	3	2
Chronic	6	6	5	3	5	3

*1 Ziehl–Neelsen stain

## Discussion

MAP is the cause of Johne’s disease that is considered as one of the important bacterial infections in camel and ruminants [26,27].

Several reports [1,20,28,29] urged that, within the Arabian Peninsula, the emerging MAP infection in dromedary camels has been dramatically increased. However, due to the ineffective efforts to isolate MAP from the infected dromedary camels, the molecular basis of Johne’s disease remained possibly elusive [20].

The isolation of MAP organism is very challenging since the difficult growth of the bacteria, and the dissimilar immune replies of the host appealed throughout the subclinical and clinical phases of MAP infection [19,30]. Interestingly, in this study, culture yielded five of thirty samples. Extensively, the diagnosis of MAP is based on fecal culture, which is measured as a gold standard method [19]. One of the most detriments of fecal culture is the sensitivity of the technique as it detects about 38–50% of diseased cases. Moreover, it consumes much time [30,31].

In this study, ZN yielded five of thirty camels. ZN is a very insensitive and unspecific test for the diagnosis of MAP, but in cases of clinical disease (as cases of this study), the sensitivity could reach 100%.

Immunodiagnostic methods were connected to guarantee the early finding of infection with MAP. In any case, the recently propelled methods were not sufficiently delicate unless they were utilized as a part of reasonable blend to accomplish a definite diagnosis [32,33]. The diagnostic sensitivity of this test is significantly impacted by different components such as infective dosage and age of the susceptible animals.

In the current study, ELISA yielded 9 of 30 samples, which might be an indicator for the unsuitability of this test to detect truly infected camels when compared to cattle. It was reported previously that ELISA assay has a diagnostic sensitivity lower than that of fecal culture, especially in subclinically infected cases [4].

Recently, the diagnostic sensitivity of MAP in fecal samples was increased obviously by the use of PCR techniques combined with nucleic acid probe [30]. This technique is carried out by amplifying the IS900 gene sequence [8,31]. In this study, PCR yielded eight of thirty samples, indicating that the sensitivity and specificity of primers used might need to re-evaluated in camels taking in consideration that these primers give a sensitivity up to 90% in cattle. These results are different from that reported by other authors [30]. The authors reported that the PCR approach is a more appropriate method than culture technique and other serological tests, as it detects non-viable and viable microorganisms in camels.

The PCR and ELISA were connected in the determination of the infection with paratuberculosis in dromedary camel [4,34]. ELISA kits that are commercially available for the identification the bovine anti-MAP antibodies were perceived reason for distinguishing the seroconverted dromedary camels [15]. In any case, ELISA was insensitive in distinguishing the anti-MAP antibodies in dromedary camels with young age [4,34]. In perspective of the varieties in the shedding designs, PCR was not realized as a proficient means in recognizing the diseased dromedary camels [4]; nevertheless, it was seen a judicious technique in identifying the irresistible ones [35].

Preceding efforts to isolate MAP from diseased camels were unsuccessful [4,8], except for Ghosh *et al*. [20], who succeeded to isolate the MAP for the first time in the kingdom of Saudi Arabia. The difficulty in culturing process of MAP is probably due to its fastidious nature. In this study, we fruitfully isolated MAP from five diseased camels where two phenotypes were detected, one was characterized by visible colonies after 16-20 weeks (slow growers), and the other phenotype was characterized by visible colonies after 6-12 weeks (fast growers). Comparable results were previously noticed for the isolation from sheep [36,37] and camel strains [1,20].

## Conclusion

From the obtained results, we concluded that the gold standard for the diagnosis of MAP is the culture method despite its limitations. Molecular diagnosis (PCR) could be a useful tool in identification of truly positive and negative camels; however, great care should be given regarding the primers specificity and sensitivity. ZN staining technique is still the most sensitive method in the identification of positive clinical cases in camels. However, sensitivity could be lower in subclinical cases. In the current study, ELISA sensitivity was higher than that of culture method, especially in the clinical cases with decreased specificity when compared to the culture method. Consequently, the ELISA test is considered unsuitable for routine diagnosis of camel MAP suspected cases. Future studies will be focused on studying the genetic diversity of camel isolates and compared it with other previously isolated strains from a variety of hosts and geographic locations.

## Authors’ Contributions

The fieldworks were done by MAS, WME, AAZ, and FMH. MAS, WME, and AMA carried out the laboratory work. WME and MAS drafted and revised the manuscript. All authors read and approved the final manuscript.

## References

[1] Salem M, El-Deeb W, Abdel-Moein K, El-Sayed A, Fayed A, Housawi F, Al-Naeem A, Zschöck M (2017). Detection of *Mycobacterium avium* subspecies *paratuberculosis* in an Egyptian mixed breeding farm and comparative molecular characterization of isolates from cattle, camels and cats a case report. Bulg. J. Vet. Med.

[2] Alluwaimi A, Hatem M, Almousa J (1999). The efficacy of gel immunodiffusion and fecal smear tests for diagnosis of ovine paratuberculosis in sheep in Saudi Arabia. Egypt. J. Immunol.

[3] Al Hajri S, Alluwaimi A (2007). The efficiency of ELISA and PCR in detecting subclinical paratuberculosis in the Saudi dairy herds. Pak. J. Biol Sci.

[4] Alhebabi A.M, Alluwaimi A.M (2010). Paratuberculosis in Camel *(Camelus dromedarius)*:The diagnostic efficiency of ELISA and PCR. Open Vet. Sci. J.

[5] Ahlstrom C, Barkema H.W, Stevenson K, Zadoks R.N, Biek R, Kao R, Trewby H, Haupstein D, Kelton D.F, Fecteau G, Labrecque O, Keefe G.P, Mckenna S.L.B, De Buck J (2015). Limitations of variable number of tandem repeat typing identified through whole genome sequencing of *Mycobacterium avium* subsp *paratuberculosis* on a national and herd level. BMC Genomics.

[6] Stabel J.R (1997). Johne's disease:A hidden threat. J. Dairy Sci.

[7] Gameel A, Ali A, Razig S, Brown J (1994). A clinico-pathological study on spontaneous paratuberculosis in camels (*Camelus dromedarius*) in Saudi Arabia. Pak. Vet. J.

[8] Alharbi K.B, Al-Swailem A, Al-Dubaib M.A, Al-Yamani E, Al-Naeem A, Shehata M, Hashad M.E, Albusadah K.A, Mahmoud O.M (2011). Pathology and molecular diagnosis of paratuberculosis of camels. Trop. Anim. Health Prod.

[9] Almujalli A, Al Ghamdi G (2012). Clinicopathological findings of paratuberculosis in camels possible steps for control strategy. Res. J. Biol. Sci.

[10] Salem M, El-Sayed A, Fayed A, Abo El-Hassan D.G (2012). Subclinical infection of paratuberculosis among camels in Egypt. J. Am. Sci.

[11] Tharwat M, Al-Sobayil F, El-Magawry S (2013). Clinicobiochemical and postmortem investigations in 60 camels (*Camelus dromedarius*) with Johne's disease. J. Camel Pract. Res.

[12] El-Deeb W, Fouda T, El-Bahr S (2014). Clinico-biochemical investigation of paratuberculosis of dromedary camels in Saudi Arabia:Proinflammatory cytokines, acute phase proteins and oxidative stress biomarkers. Pak. Vet. J.

[13] Hereba A, Hamouda M, Al-Hizab F (2014). Johne's disease in dromedary camel:Gross findings, histopathology and PCR. J. Camel Pract. Res.

[14] Sweeney R, Jones D, Habecker P, Scott P (1998). Interferon-gamma and interleukin 4 gene expression in cows infected with *Mycobacterium paratuberculosis*. Am. J. Vet. Res.

[15] Alluwaimi A.M (2015). Paratuberculosis infection in camel (*Camelus dromidarius*):Current and prospective overview. Open J. V. Med.

[16] Cocito C, Gilot P, Coene M, De Kesel M, Poupart P, Vannuffel P (1994). Paratuberculosis. Clin. Microbiol. Rev.

[17] Valentin-Weigand P, Goethe R (1999). Pathogenesis of *Mycobacterium avium* subspecies *paratuberculosis* infections in ruminants:Still more questions than answers. Microbes Infect.

[18] Stevenson K, Hughes V.M, De Juan L, Inglis N.F, Wright F, Sharp J.M (2002). Molecular characterization of pigmented and nonpigmented isolates of *Mycobacterium avium* subsp *paratuberculosis*. J. Clin. Microbiol.

[19] Salem M, Heydel C, Elsayed A, Ahmed A, Zschöck M, Baljer G (2013a). *Mycobacterium avium* subspecies *paratuberculosis*:An insidious problem for the ruminant industry. Trop. Anim. Health Prod.

[20] Ghosh P, Hsu C, Alyamani E.J, Shehata M.M, Al-Dubaib M.A, Al-Naeem A, Hashad M, Mahmoud O.M, Alharbi K.B, Al-Busadah K, AL-Swailem A.M, Talaat A.M (2012). Genome-wide analysis of the emerging infection with *Mycobacterium avium* subspecies *paratuberculosis* in the Arabian camels (*Camelus dromedarius*). PLoS One.

[21] Manning E.J, Collins M.T (2001). *Mycobacterium avium* Subsp *Paratuberculosis*:Pathogen, pathogenesis and diagnosis. Rev. Sci. Tech.

[22] Sellon R (2008). Rectal Scrape Cytology. Gastrointestinal Cytology. CVC Proceeding.

[23] Bull T.J, McMinn E.J, Sidi-Boumedine K, Skull A, Durkin D, Neild P, Rhodes G, Pickup R, Hermon-Taylor J (2003). Detection and verification of *Mycobacterium avium* subsp *paratuberculosis* in fresh ileocolonic mucosal biopsy specimens from individuals with and without Crohn's disease. J. Clin. Microbiol.

[24] Bannantine J.P, Baechler E, Zhang Q, Li L, Kapur V (2002). Genome scale comparison of *Mycobacterium avium* subsp *paratuberculosis* with *Mycobacterium avium* Subsp. Potential diagnostic sequences. J. Clin. Microbiol.

[25] Salem M.A, Zeid A, Hassan D, El Sayed A, Zschoeck M (2005). Studies on Johne's disease in Egyptian cattle. J. Vet. Med. B. Infect. Dis. Vet. Public Health.

[26] Crawford C.G, Ziccardi H.M, Gonzales J.B (2006). *Mycobacterium avium* subspecies *paratuberculosis* and *Mycobacterium avium* Subsp. Avium infections in a tule elk (*Cervus elaphus nannodes*) herd. J. Wildl. Dis.

[27] Behr A.M, Collins M.D (2010). Paratuberculosis, Organism, Disease, Control.

[28] Paling R.W, Waghela S, Macowan K.J, Heath B.R (1988). The occurrence of infectious-diseases in mixed farming of domesticated wild herbivores and livestock in Kenya.2. bacterial diseases. J. Wildl. Dis.

[29] Al-Hizab F.A (2010). Johne's disease in one-humped camels (*Camelus dromedarius*) in Saudi Arabia. J. Camel Pract. Res.

[30] Haghkhah M, Derakhshandeh A, Jamshidi R, Moghiseh A, Karimaghaei N, Ayaseh M, Mostafaei M (2015). Detection of *Mycobacterium avium* subspecies *paratuberculosis* infection in two different camel species by conventional and molecular techniques. Vet. Res. Forum.

[31] Stabel J.R, Bosworth T.L, Kirkbride T.A (2004). A simple, rapid, and effective method for the extraction of *Mycobacterium paratuberculosis* DNA from fecal samples for polymerase chain reaction. J. Vet. Diagn. Invest.

[32] Paolicchi F, Zumárraga M, Gioffre A, Zamorano P, Morsella C, Verna A, Cataldi A, Romano M (2003). Different methods for the diagnosis of *Mycobacterium avium* subsp *paratuberculosis* in a dairy cattle herd in Argentine. J. Vet. Med. B. Infect. Dis. Vet. Public Health.

[33] Collins D.M, Stephens D.M, De Lisle G.W (1993). Comparison of polymerase chain 16 reaction tests and fecal culture for detecting *Mycobacterium paratuberculosis* in 17 bovine faeces. Vet. Microbiol.

[34] Alluwaimi A (2008). The efficiency of bovine ELISA in detection of the *Mycobacterium avium* subspecies *paratuberculosis*(MAP) infection in Camel (*Camelus dromedaries*) at different ages. J. Camel Pract. Res.

[35] Nielsen S.S, Toft N (2008). Antemortem diagnosis of paratuberculosis:A review of accuracies of ELISA, interferon-gamma assay and faecal culture techniques. Vet. Microbiol.

[36] Whittington R.J, Marsh I, Mcallister S, Turner M.J, Marshall D.J (1999). Evaluation of modified BACTEC 12B radiometric medium and solid media for culture of *Mycobacterium avium* subsp *paratuberculosis* from sheep. J. Clin. Microbiol.

[37] De Juan L, Alvarez J, Romero B, Bezos J, Castellanos E (2006). Comparison of four different culture media for isolation and growth of Type II and Type I/III *Mycobacterium avium* subsp *paratuberculosis* strains isolated from cattle and goats. Appl. Environ. Microbiol.

